# Current understanding of the gut microbiota shaping mechanisms

**DOI:** 10.1186/s12929-019-0554-5

**Published:** 2019-08-21

**Authors:** Cherng-Shyang Chang, Cheng-Yuan Kao

**Affiliations:** Immunology Research Center, National Health Research Institutes, Zhunan, Miaoli 35053 Taiwan

**Keywords:** Gut microbiota, Intestinal epithelium, Barrier, Microbiota shaping

## Abstract

Increasing evidences have shown strong associations between gut microbiota and many human diseases, and understanding the dynamic crosstalks of host-microbe interaction in the gut has become necessary for the detection, prevention, or therapy of diseases. Many reports have showed that diet, nutrient, pharmacologic factors and many other stimuli play dominant roles in the modulation of gut microbial compositions. However, it is inappropriate to neglect the impact of host factors on shaping the gut microbiota. In this review, we highlighted the current findings of the host factors that could modulate the gut microbiota. Particularly the epithelium-associated factors, including the innate immune sensors, anti-microbial peptides, mucus barrier, secretory IgAs, epithelial microvilli, epithelial tight junctions, epithelium metabolism, oxygen barrier, and even the microRNAs are discussed in the context of the microbiota shaping. With these shaping factors, the gut epithelial cells could select the residing microbes and affect the microbial composition. This knowledge not only could provide the opportunities to better control many diseases, but may also be used for predicting the success of fecal microbiota transplantation clinically.

## Introduction

**Fig. 1:**
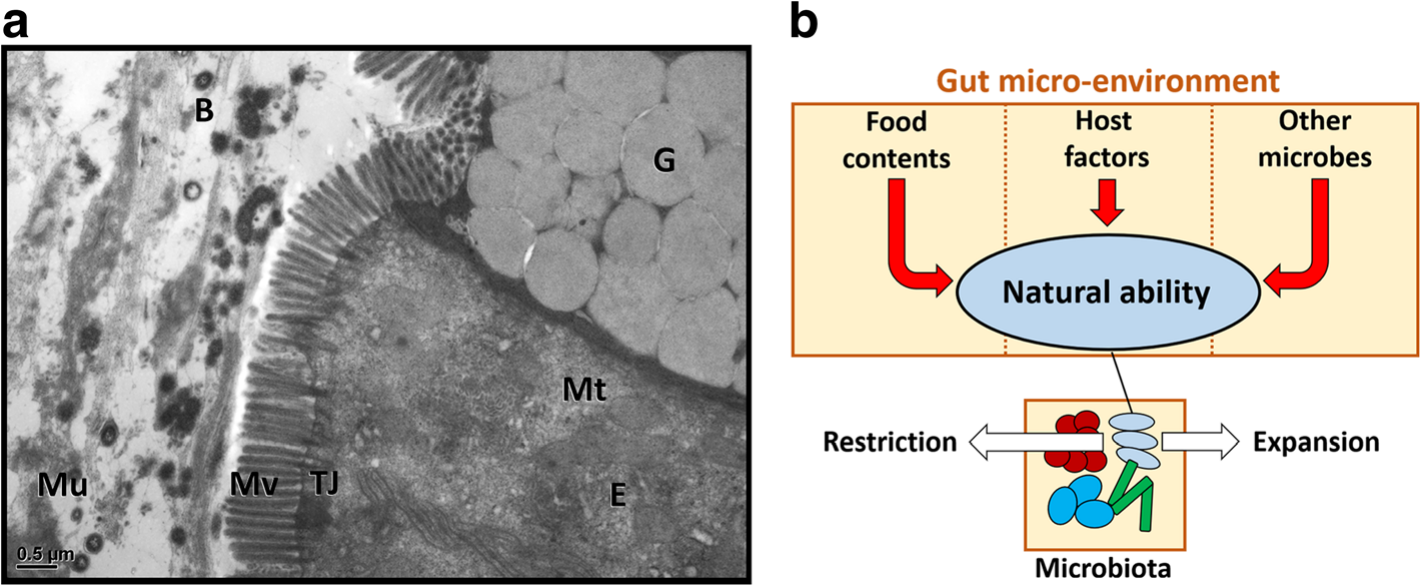
The micro-environment in the gut lumen determines the gut microbiota composition. **a** Transmission electron microscope image of mouse colon displays the spatial relation of microbes and gut epithelium. G, goblet cells; E, epithelial cells; B, Bacteria; TJ, tight junction; Mu, mucus; Mv, microvilli; and Mt, mitochondria. Scale bar = 0.5 μm. **b** The gut micro-environment possess a variety of stimulators originated from the digested food, host and other microbes. The sum of all these stimulators provides the selection force to shape the gut microbiota. Meanwhile, different responses from diverse microbes to the stimulations also affect the microbiota composition

The last human organ, a separate organ, a forgotten organ, a new organ or a missing organ––all of these appellations point out the existence of the gut microbiota and emphasize its importance [1–5]. The change of gut microbial composition not only has been shown associated with the intestinal diseases such as inflammatory bowel disease (IBD) [6–8], irritable bowel syndrome (IBS) [9], and colorectal cancer (CRC) [10], but also linked to the non-intestinal diseases such as allergy [11, 12], asthma [13], obesity [14, 15], nonalcoholic fatty liver [16], cardiovascular diseases [16, 17] and neuro-psychiatric diseases [18, 19]. These diseases can be often attributed to the altered microbiota, which would be further referred to as dysbiosis or dysregulation of microbiota. However, the words “dysbiosis” and “dysregulation” are biased from the host’s aspects. The ecological change of gut microbes is merely a consequence of microbes in response to the external stimulations according to their natural ability. Different ability such as metabolic machinery, sensing-response system, oxygen resistance, thermal tolerance, and even the virulence factors within microbes result in the diverse microbial populations under the various selection force from external micro-environment (Fig. 1).

The hypothesis that host factors could directly affect the gut microbiota is mainly supported by a series of studies in twins [20–27]. As early as 2001, Zoetendal et al. used the denaturing gradient gel electrophoresis (DGGE) fingerprinting to analyze the bacterial composition in twins. They found that the similarity of gut bacteria in the monozygotic (MZ) twins were significantly higher than those in genetically unrelated individuals, indicating that the host factors have important impact on regulation of the gut bacterial composition in adult human [27]. In 2005, Stewart et al. performed the temporal temperature gradient gel electrophoresis (TTGE) fingerprinting and demonstrated that the MZ twins have higher similarity of their gut bacterial population as compared with the dizygotic (DZ) twins [20]. Turnbaugh et al. and Yatsunenko et al. subsequently performed the 16 s rRNA gene sequencing and reported that MZ twins have slightly more similar gut microbiomes as compared with DZ twins, despite the differences have no statistical power [21, 22]. Hansen et al. specifically demonstrated that the concordance rate for carriage of the methanogen *Methanobrevibacter smithii* is higher for MZ twins than DZ twins [23]. In 2014, Goodrich et al. performed a larger 16 s rRNA gene sequencing of twins, and the difference of gut microbiome between MZ twins and DZ twins reached statistical significance [24]. Importantly, they identify some microbial taxa whose abundances were affected by host genetics, demonstrating the hypothesis of “microbiome heritability”. Extended from this study, Goodrich et al. performed a project that tripled the sample size and successfully found out several host genes associated with microbiome shaping [25]. In 2016, Xie et al. performed the first shotgun metagenomic analysis of twins’ microbiome and validated the impacts of host on the gut microbiota, though their evidence also has no statistical power due to a relatively small size of cohort [26]. In addition to the twin studies in human, Benson et al. demonstrated that the host genetics shapes the individual microbiome diversity in mouse [28]. Totally 18 quantitative trait loci (QTL) were identified to be associated with various bacterial taxa in the mouse gut. Moreover, a variety of knockout studies of several of genes in mice showed the link between host genes and gut microbiota. Together, these evidences have stressed the importance of host factors in modulation of gut microbiota. However, how host genes modulate the gut microbiota remains largely unknown [29, 30]. To know how microbiota is shaped in the gut, we review the current studies and discuss what host factors could be involved in the regulation of microbiota. Since a number of articles have already discussed the effects of microbiota on the host [29], these effects are beyond the scope of this review. Instead, here we focus on the modulating direction from the host toward the microbiota, particularly on the roles of epithelium, the frontier with gut microbiota, in the gut microbiota shaping mechanisms.

### The epithelium-associated factors involved in gut microbiota shaping

**Fig. 2:**
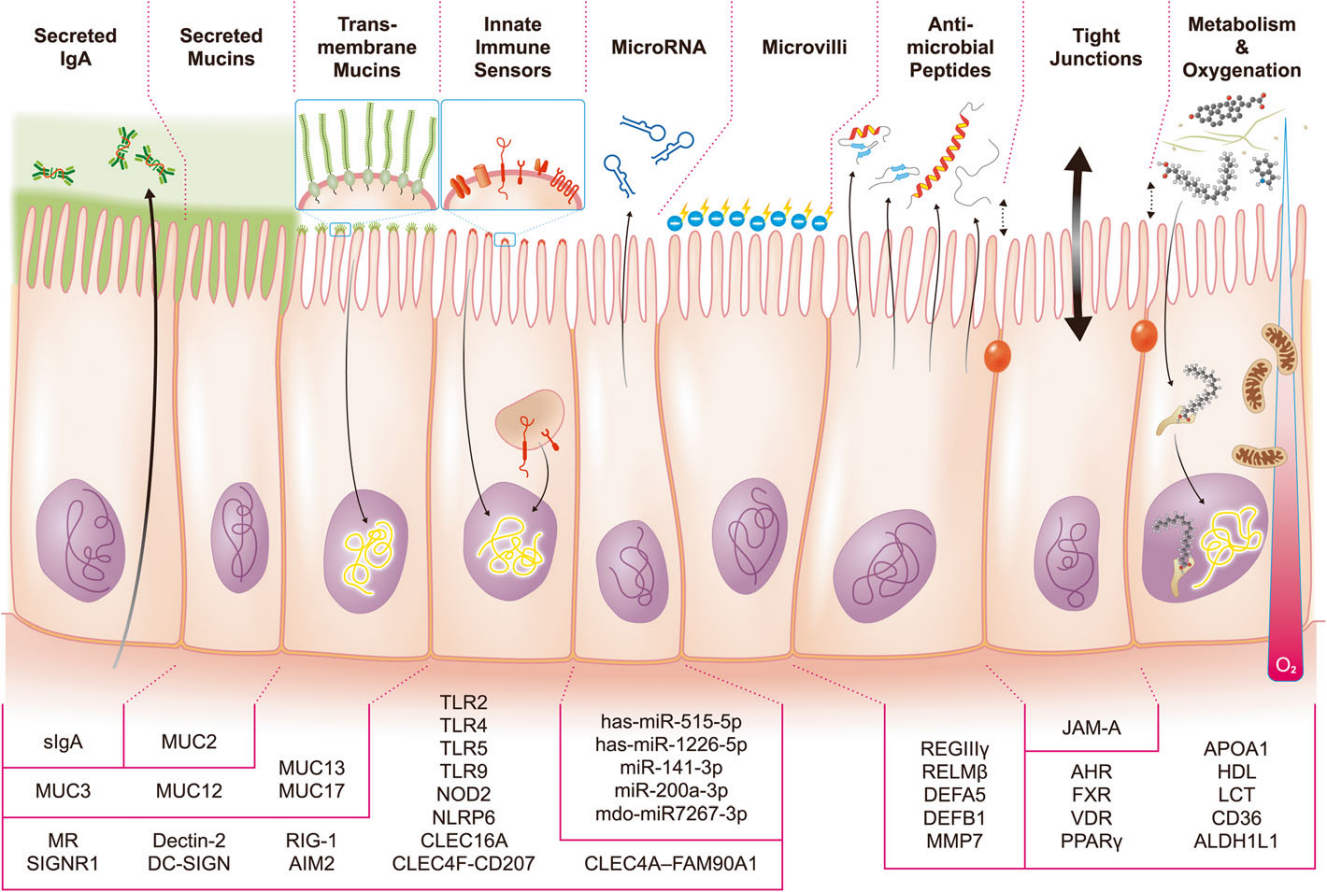
The epithelium-associated factors shape the microbiota in the gut. The gut epithelial cells act as the frontline mediators affecting the establishment of commensal microbiota via a number of shapers

Gut is a complex organ composed of multilayer of tissues, in which gut epithelia act as the frontline in response to the direct and indirect contact of luminal microbes. Herein we collected the current evidences to show the impact of epithelium-associated factors on gut microbiota (Fig. 2). The role of epithelium-associated factors including the epithelial innate immune sensors, anti-microbial peptides (AMPs), mucus barrier, secretory IgAs (sIgAs), epithelial microvilli, epithelial tight junctions, epithelial metabolism, oxygen barrier, and even the microRNA in the microbiota shaping were discussed as follows:

### Innate immune sensors

Accumulating evidences have shown the role of innate immunity of gut epithelium in shaping microbiota [29]. The enterocytes are known to express the pattern recognition receptors (PRRs) for sensing the microbe-associated molecular patterns (MAMPs) and thereby promoting the immune responses including production of anti-microbial peptides, transportation of sIgAs and recruitment of immunocytes [31]. PRRs can be classified into five families: Toll-like receptors (TLRs), C-type lectin-like receptors (CLRs), nucleotide-binding oligomerization domain (NOD)-like receptors (NLRs), retinoic acid-inducible gene-I (RIG-I)-like receptors (RLRs), and recently designated absent-in-melanoma (AIM)-like receptors (ALRs) [29, 32]. These PRRs determine the sensing-response system of the host, and play critical roles in microbiota shaping.

#### TLRs

TLR2 deficient mice showed an alteration of gut microbiota with a higher abundance of *Helicobacter* [33]. While no direct evidence showed that the TLR2 in epithelial cells affects specific bacteria taxa, TLR2 in T cells has been proved to help the colonization of commensal *Bacteroides fragilis* in the gut [34]. The mice with intestinal epithelium-overexpression of TLR4 displayed higher abundances of *Fusobacteria* and *Proteobacteria* and lower abundances of *Firmicutes* in the colonic mucosa than their littermate wild-type controls [35]. Another study revealed TLR4 knockout in mice decreased the abundance of *Bacteroidetes* [36]. Furthermore, alteration of gut microbial composition in particular the abundances of the *Bacteroidetes* and *Lachnospiraceae* has also been reported in mice deficient in TLR5 [37]. TLR9 knockout mice harbored slightly lower levels of *Enterobacteria* and *Bacteroides*, whereas levels of *Clostridium leptum* were higher compared to wild-type mice. Notably, *Bifidobacteria* were absent in the TLR9 knockout mice [38].

#### NLRs

An earlier study has shown that NOD2 knockout mice have down-regulated expression of α-defensins and were more susceptible to *Listeria monocytogenes* infection [39]. Following studies reported that NOD2 knockout mice harbor a higher amount of *Bacteroides*, *Firmicutes* and *Bacillus* in the terminal ileum compared with their littermate wild-type controls [40, 41]. NLRP6 inflammasome-deficient mice exhibit both qualitative and quantitative alterations in many taxa, including increased abundances of *Prevotellaceae* and TM7, and reductions of genus *Lactobacillus* in the *Firmicutes* phylum compared with wild-type mice [42]. Recently, polymorphisms in NOD2 gene were found to be associated with changes in the levels of *Enterobacteriaceae* in humans [43]. Polymorphisms in the NOD1 gene were also found to be associated with the abundance of *Enterobacteria* [44].

#### CLRs

The CLRs have being known to be critical in anti-fungal immunity, but relatively rare report has described about whether these receptors are involved in gut bacterial recognition and microbiota shaping [45]. Mannose receptors (MR), SIGNR1 and Dectin-2 have been demonstrated to recognize the bacterial capsular polysaccharides derived from *Streptococcus pneumoniae* [46], but this bacterium is not usually found in the gut. *Lactobacillus reuteri* and *Lactobacillus casei* have been demonstrated to interact DC-SIGN and induce regulatory T-cells, and the surface layer A protein (SlpA) on the surface of *Lactobacillus acidophilus* has been identified as a ligand of this CLR [45]. Recently, two genome-wide association studies (GWAS) discovered some gut microbiota-associated CLRs, including the CLRs CLEC4F-CD207, CLEC4A-FAM90A1 and CLEC16A [44, 47].

#### RLRs

RIG-1 has been demonstrated to be constitutively expressed in gut epithelial cells and it is previously known to play a crucial role not in anti-viral responses as the intracellular receptor for recognition of double-stranded RNA from viruses [48, 49]. Notably, RIG-1 has been demonstrated to sense not only viral but also bacterial RNA to induce the production of type I interferons [50, 51]. A recent study by Zhu et al. showed that the Rig-I knockout mice display an altered microbiota in comparison with wild-type mice and they further found that this microbial change could be linked to the down-regulation of IgA, REGIIIγ and PD-1 [52].

#### ALRs

AIM2, which belongs to ALRs family, is known to recognize intracellular bacterial DNA [53–55], and is involved in the mediation of antimicrobial peptides such as C-type lectins (REGIIIβ and REGIIIγ), calprotectin (S100A8 and S100A9) and lipocalin 2 (Lcn2) in gut epithelial cells [56]. Aim2 has been demonstrated to be required for the recognition of invasive pathogens such as *Francisella tularensis* in the cytoplasm [57]. Importantly, Hu et al. demonstrated that the abundances of *Escherichia coli* and family *Enterobacteriaceae* were significantly higher in *Aim2* knockout mouse feces as compared with those in the wild-type mice, suggesting that the DNA sensor ALRs also play a role in regulation of microbial ecology in the gut luminal space [56].

### Anti-microbial peptides (AMPs)

Many evidences have shown the importance of AMPs in shaping gut microbiota. The REGIIIγ, a secreted C-type lectin, has been proved to target the bacteria through interacting with peptidoglycan carbohydrate [58]. The knockout of resistin-like molecule β (RELMβ), a cytokine that mediates the expression of REGIIIγ, impacts the abundance of *Bacteroidetes*, *Firmicutes* and *Proteobacteria* [59]. The mice transgenic for DEFA5, a human α-defensin, showed a lower abundance of *Firmicutes* and the higher percentage of *Bacteroidetes* as compared with non-transgenic control [59]. The mice lacking MMP7, an enzyme required for the processing of mouse α-defensin, displayed a significantly higher abundances of *Firmicutes* and a significantly lower abundances of *Bacteroidetes*, when compared with the wild-type mice. In addition, β-defensins such as DEFB1 have also been shown to have bactericidal effects against the gram-positive commensals of *Bifidobacterium* and *Lactobacillus* [60, 61].

### Epithelial mucus barrier

Enterocytes are known to express the transmembrane mucins for the development of “glycocalyx” on the apical surface of microvilli [62–64]. The transmembrane mucins such as MUC3, MUC12, MUC13 and MUC17 functionally form the protective brush that may act as the diffusion barrier in the gut, maintain the integrity of the surface epithelial layer, and limit the passage of large molecules in the lumen [63, 65]. The cytoplasmic domains of MUC3, MUC12 and MUC17 are able to interact with different PDZ-proteins, thereby regulating the membrane channels and signal proteins [63]. Thus, the transmembrane mucins can act as the protective barrier or luminal sensor for gut immunity, and could be involved in the regulation of gut microbiota.

Besides the transmembrane mucins, the goblet cells secrete the gel-forming mucins into the lumen for the construction of mucus wall. In colon, the mucus wall can be further divided into two layers: the inner firm layer that forms a coat for segregating the microbes and the outer loose layer that provides a habitat for residing microbes [65, 66]. Gut microbiota has been reported to be altered by the deletion of *Muc2* gene in mice [67]. The Muc2 knockout mice gut microbiome displayed a more enriched *Firmicutes* and decreased *Bacteroidetes* at phylum level. Moreover, increased levels of *Desulfovibrio*, *Escherichia*, *Akkermansia*, *Turicibacter*, *Erysipelotrichaceae* and *Ruminococcaceae* and decreased levels of *Lactobacilli* and *Lachnospiraceae* were observed in Muc2 deficient mice. This result could be attributed to the diverse ability of different microbes to degrade and utilize the mucus [68, 69]. Muc2 and other mucins are modified with complex and unique glycans that could be cleaved by exoglycosidases from specific bacteria. Some bacterial species have lots of catabolic glycosidic enzymes to degrade complex mucus glycans as a carbon source. Therefore, the glycans on the mucus also play a role in the regulation of gut microbiota.

In sum, the gut epithelial cells build a mucus barrier composed of transmembrane mucins/epithelial glycocalyx and secreted gel-forming mucins/mucus wall. The mucus layer of gut provides a space for host-microbes interplay or communication. Further study is required to elucidate the effect of specific mucins or its glycans on the composition of microbiota.

### Secretory IgA (sIgA)

In the gut, sIgAs are produced by plasma cells in the lamina propria and transported through the enterocytes into the lumen, where they interact with mucins and bacteria in the outer mucus layer [70, 71]. The reduction of sIgA levels in Rig-1 knockout mice and cytokine lymphotoxin (LT)-α knockout mice has been reported to induce the changes of gut microbiota [52, 72]. Some evidence also showed that the sIgAs in inhibitory co-receptor programmed cell death-1 (PD-1) knockout mice have reduced bacteria-binding capacity, which causes the alteration of gut microbiota [73]. Recently, the role of IgA in regulating microbial ecology was also confirmed in humans with IgA deficiency [74]. Therefore, the sIgA is critical for shaping gut microbiota and the control of gut ecology homeostasis.

The IgA receptors such as immunoglobulin receptor (pIgR), CD71, and CD89 identified on the epithelial cells could also help the enterocytes bind for the clearance sIgA-bound microbes [62]. The studies showed that sIgAs help host not only in the clearance of pathogens but also the anchoring of commensals in mucus. Specific recognition of sIgA has been proved to help commensal *Bacteroides fragilis* adherence to gut epithelial cells [75]. sIgA has also been shown to enhance adherence of *Escherichia coli*, *Bifidobacterium lactis* and *Lactobacillus rhamnosus* to epithelial cells [76, 77], revealing that the microbes may also benefit from sIgA to build up a mucosal microbial community. sIgA-coated bacteria from healthy humans are found to protect mice from diseases [78]. Similarly, the breastmilk-derived sIgA is also demonstrated on a role in shaping gut microbiota [11]. Together, these evidences show that sIgAs have diverse binding affinity with different bacteria, which in turn, provide a selection pressure for shaping the microbial composition.

### Epithelial microvilli (electrostatic barrier)

Each enterocyte contains thousands of microvilli, which form the brush border to increase the apical surface area, and then facilitate the absorption of nutrients and defense against luminal microbes [79]. The molecular motors within the microvilli are able to send the vesicles packed with gut enzymes out for digestion [80]. Importantly, epithelial microvilli were demonstrated to establish an electrostatic barrier for resisting microbial adhesion [81]. Unlike the attractive forces caused by the epithelial IgA, mucus and receptors, the epithelial microvilli exhibit the negative charge on the luminal surface which provides a repulsive force against the adhesion of mucosa-associated microbes. The surface negative charges of diverse microbes are different; therefore, the electrostatic force of microvilli is also one of shaping factors for microbiota.

### Epithelial tight junction (physical barrier)

The gut epithelial cells link together by forming intercellular tight junctions (TJ) to provide a physical barrier, which limits digested food and gut microbes freely coming across into deeper tissue [82–84]. Studies have showed that gut commensals or probiotics can induce TJ protein expressions and help the host decrease paracellular permeability [85, 86], and yet other studies have showed that commensals can also secret protease to degrade TJs [87]. Some pathogens are demonstrated to disrupt the TJ complex via instigating the enterocytes to down-regulate or internalize the TJ proteins [88, 89]. Although some studies have showed various effects of diverse microbes on the host epithelial TJ expression, the direct evidence showing that TJ shapes gut microbiota is still lacking. Therefore, it is more likely that the disruption of epithelial TJ allows the luminal microbes or their components to activate the immunocytes in the lamina propria, which would indirectly contribute to the shaping of microbiota. Interestingly, one recent study showed the potential of TJ protein in regulating microbiota. The junctional adhesion molecule A (JAM-A) knockout mice displayed a significant increase of *Desulfovibrionaceae* and decrease of *Akkermansia* in their gut microbiota [90]. Of note, this phenomenon was only observed in the mice fed with a diet high in saturated fat, fructose and cholesterol but not the mice fed with normal diet, suggesting that the microbiota shaping effect of TJ may be difficult to be observed in basal state. Certain stress models could be required in the testing the roles of TJ protein in the regulation of gut microbiota.

### Epithelial metabolism and oxygen barrier

The host and gut bacteria share the nutrients from the same digests in the gut, and therefore the host-microbe interaction is indeed a competition, and the performance of host to utilize the nutrients could consequently affect the population of the opponent microbes. For example, the mice lacking APOA1, a major component of high-density lipoprotein (HDL), harbored a decreased abundance of *Erysipelotrichaceae* and increased abundance of *Lachnospiraceae* [91]. A 16 s rRNA-based study has showed that the polymorphism of LCT, a gene encoding lactase for the hydrolysis of lactose, can be linked with the abundance of *Bifidobacterium* [25]. The genus *Blautia* has been found to be associated with the polymorphisms of CD36, a gene involved in the absorption of long-chain fatty acid in the gut [25]. The polymorphisms of ALDH1L1, a gene encoding for an aldehyde dehydrogenase involved in the formate oxidation, has also been linked with the order SHA-98, a member of the *Christensenellaceae consortium* [25]. Thus, the metabolites utilization of host could impact the bacteria on their composition in the gut.

Several metabolite sensors expressed in the gut epithelia are demonstrated to be activated by binding with the microbe-derived metabolites and therefore could be involved in gut microbiota shaping [92]. For instance, the dietary tryptophan can be degraded by gut commensals such as *Lactobacilli* into indole derivatives, and as the agonists of the aryl hydrocarbon receptor (AHR) [92, 93]. The small intestine of wild-type mice fed with diet depleted of AHR ligands harbored lower levels of *Firmicutes* and higher levels of *Bacteroidetes* than the mice fed with the diet contained AHR ligands [94]. Increased levels of phyla *Bacteroidetes* were also observed in the small intestine and colon of AHR deficient mice, suggesting that the AHR is not only a sensor but also a regulator of gut microbiota [94, 95]. Apart from AHR, farnesoid X receptor (FXR), a nuclear receptor that is known to be activated by secondary bile acids digested by commensals, is also associated with alteration of gut microbiota. Decreased levels of *Firmicutes* and increased levels of *Bacteroidetes* were found in FXR deficient mice compared with wild-type mice after 10-week feeding of high-fat diet [96]. The secondary bile acids are also demonstrated to directly activate vitamin D receptor (VDR) [97, 98]. VDR deficient mice showed increased levels of *Clostridium* and *Bacteroides* and decreased levels of *Lactobacillus* in the feces. Study of both human and mice gut microbiota indicated that VDR influences individual bacterial taxa such as *Parabacteroides* [47]. In addition, other microbe-derived metabolites such as butyrate and propionate are proved to activate nuclear receptors such as peroxisome proliferator activated receptor gamma (PPARγ) [99, 100], which are known to repress inflammation and increase the production of β-defensins [101]. However, while those and many other nuclear receptors have been found to serve as metabolic sensors for microbiota shaping, further studies are required to elucidate their roles in the epithelial cells and immunocytes in the gut, regardless of whether these factors are already proved to be expressed in the epithelial cells [92].

In addition to the metabolite utilization, a concept of the oxygen metabolism and oxygen barrier shaping gut microbiota composition has been recently proposed [102]. This concept is originated from the “oxygen hypothesis” proposed by L. Rigottier-Gois, who described that the IBD patients share a similar gut microbiome pattern such as decreased obligate anaerobes (*Faecalibacterium prausnitzii*) and increased facultative anaerobes (*Enterobacteriaceae*) [103]. In IBD, an increase in the luminal oxygen level could be resulted from the leakage of epithelium, provoking the release of hemoglobin carrying oxygen in the mucus layer where the gut bacteria reside. The increased oxygen level disrupts the epithelial anaerobiosis. This could further provide an ecological selective advantage to facultative anaerobes or potentially aerobes, which allows them to be more competitive to expand. For instance, the aerobic expansion of pathogenic bacteria such as *Salmonella* was found under the disruption of anaerobiosis [104]. Importantly, it was found that the increase of the luminal oxygen level is not only resulted from the leakage of physical barrier that controls the paracellular pathway but also caused by the increased anaerobic glycolysis that reduces the oxygen consumption in the transcellular pathway, especially in the colonic epithelia. Unlike the small intestinal epithelia which prefer the usage of glucose and glutamine [105], the matured colonic epithelia mainly generate energy by oxidizing the short-chain fatty acid such as butyrate, which could render the mucosal surface hypoxic [106, 107]. However, if colonic epithelial cells switch to a preferred use of glucose, the remaining oxygen could diffuse into the intestinal lumen, and eventually cause the expansion of facultative anaerobes such as *Enterobacteriaceae.* Indeed, the newborn infants have an aerobic intestine at birth [108]. The relatively higher level of oxygen in the newborn intestinal tract favors the appearance of facultative anaerobes such as *Enterobacteriaceae*, *Enterococcus*, and *Streptococcus*. These early colonizers consume the available oxygen and thereby create an anaerobic micro-environment in the gut and facilitate the establishment of obligate anaerobes such as *Bifidobacterium*, *Clostridium*, *Bacteroides*, *Veillonella*, *Eubacterium*, and *Ruminococcus* species. All these evidences support that the oxygen level can as a shaper of host in regulation of gut microbiota [106].

In sum, both the metabolic energy flow and development of oxygen barrier on the host side have great influence on the gut microbial composition. Of note, all the impacts of host metabolism on gut microbiota relied on the precondition of the formation of physical barrier discussed here. The development of intercellular junctions is the key factor for gut to establish a boundary that limits the metabolites inflow and oxygen outflow.

### microRNA

MicroRNAs are 18–23 nucleotides in length non-coding RNAs. So far, it is known that microRNAs could exist extracellularly and appear in body fluids [109]. Studies have also found RNA in human stool, and fecal microRNAs are considered as biomarkers of intestinal diseases such as colitis and dysbiosis [110, 111]. Importantly, studies also suggest that microRNAs produced by the host’s intestinal epithelial cells could participate in shaping the microbiota [110, 112, 113]. In 2016, Liu et al. reported that the human microRNA such as miR-101, hsa-miR-515-5p, miR-876-5p, hsa-miR-325 and hsa-miR-1253 could affect gene expression of the anaerobic species *Fusobacterium nucleatum*; hsamiR-4747-3p, hsa-miR-1224-5p, hsa-miR-1226-5p and hsa-miR-623 could change gene expression of the facultative anaerobic *E. coli* [110]. They further demonstrated that the has-miR-515-5p and has-miR-1226-5p could promote the growth of *Fusobacterium nucleatum* and *E. coli*, respectively. Moreover, four microRNAs, let-7b-3p, miR-141-3p, miR-200a-3p, and mmu-1224-5p, have been proved to be constitutively expressed in murine intestinal epithelial cells. Moloney et al. further validated these murine microRNA candidates, and found that the abundances of the phyla *Bacteroidetes* and *Firmicutes* were correlated with the level of miR-141-3p, and phyla *Actinobacteria*, *Bacteroidetes*, *Cyanobacteria*, *Firmicutes* and *Proteobacteria* were significantly correlated with miR-200a-3p level [113]. Interestingly, in addition to the animal microRNAs, the plant-derived microRNA such as ginger microRNA mdo-miR7267-3p has been demonstrated to affect the gut microbiota [114]. While the molecular mechanisms behind these phenomenons still remain largely unknown, these evidences do demonstrate that the host can specifically affect the microbes, and regulate the gut microbial compositions.

### Potential of microbiota shaping factors applied in intestine-on-a-chip

The host-microbes interactions are indeed bidirectional. While most of the mainstream microbiota studies focus on the effect of microbes on the host cells, we emphasize the importance of the roles of host in shaping the microbiota in this review. Nevertheless, in order to get a thoroughly understanding of this bidirectional communication, a proper experimental model is required. In the past, it is hard to co-cultivate the gut microbes and host living epithelium for a very long period because the overgrowth of microbes may disturb the host-microbes balance and the microbe-derived organic acids could interfere the host cells. The difference in nutrition or oxygen demand between the host cells and microbes also limits the ability of researchers to study the microbiota shaping mechanism. Recently, the development of intestine-on-a-chip model by using the microfluidic technique provides a solution for counteracting these problems [115]. For example, the intestine-on-a-chip could supply a continuous flow to remove the microbe-derived organic acids and the non-adherent bacteria during co-cultivation [116]. The host cells and microbes can be cultivated at different locations or diverse chambers within a chip, and therefore the host cells and bacteria can be cultivated under different oxygen concentrations at the same time in the same system [117–119]. The intestine-on-a-chip can be fabricated with villi-like structure to mimic the intestinal surface [120–122]. However, so far the intestine-on-a-chip studies were only used to test the effect of microbes on the host cells.

As we have discussed in this review, the host factors should not be ignored. The intestine-on-a-chip model could be used to examine the effect of hosts on individual microbe or microbiota. The host cells with overexpression or knockout of gene can be cultivated in the intestine-on-a-chip to validate the host genetic effects on the microbes. The intestine-on-a-chip has been proposed to be used for prediction of the efficacy of fecal microbiota transplantation (FMT) clinically [123], and the intestine-on-a-chip might also be used for shaping the patient’s microbiota in the future.

Several limitations of intestine-on-a-chip for investigating microbiota shaping factors and for predicting FMT success in patient still need to be solved. For example, while the intestinal cell lines such as Caco-2 and HT-29 have been widely used for intestine-on-a-chip, the property of the cancer cells is different from the normal intestinal cells. It is also important to note that the gut epithelium is composed of multitype rather than a single type of cell. Recently, Kasendra et al used organoids technique in the chip and evidently addressed these issues [124]. They isolated the intestinal stem cells from normal regions of human intestinal biopsies, expanded and differentiated the epithelial cells by developing the 3D intestinal organoids, and successfully lined the heterogenous epithelial cells on the 2D surface of the chip. Importantly, this organoids-on-a-chip system can expose the apical side of the epithelium rather than enclosing it to form a separate chamber, allowing the researchers to study the host-microbes interactions more easily. However, so far it still costs a significant amount of time and money for the development of organoids, which would hinder the practice in clinic [125]. Furthermore, factors such as age, gender, and geographic region are known to affect microbiota or host gene expression [126, 127]. Therefore, a high-throughput intestine-on-a-chip system is required to get a sufficient amount of information to establish a reliable database for FMT prediction. Nevertheless, it is worth paying attention to the development of next-generation intestinal chip, especially in utilization for the study of microbe-host interactions.

### Clinical insights from microbiota shaping factors into FMT recipient and super-donor

FMT, a modish approach to restore the gut microbiota homeostasis by transferring fecal microbiota from healthy donors to patients, has been used for recurrent and refractory *Clostridium difficile* infections (CDIs), yet 12.4% of the CDI patients still suffer the FMT failure [128]. Recently, the first case of FMT death was reported. One adult died due to the infection of undetected extended-spectrum beta-lactamase (ESBL)-producing *Escherichia coli* from the donor. This unfortunate case highlights the importance of donor selection before practice of FMT, and emphasizes the need of prediction of FMT effects on recipient. In fact, the success rate of FMT still has room for improvement in other type of intestinal disease such as ulcerative colitis (UC). According to the results of the recent clinical trials, there are merely 24–30% of UC patients were in remission after FMT [129–131]. While these randomized control trials showed that FMT has higher remission rate for treating UC compared with placebo control, the insufficient rate of success indeed casts a shadow on the practice of FMT. Moreover, a recent study showed that the UC patients with antibiotic-dependent pouchitis (ADP) have low success rate (17%) of FMT due to the failure of engraftment [132]. The authors concluded that this failure could be due to the factors including donor selection, dose and frequency of FMT, and the microenvironment in the ileal pouch of patient. Thus, to increase the success rate of FMT, thoroughly understanding of the factors from both donor and recipient is required.

The term “super-donor” has been recently used to describe some donors whose stool could confer significantly more successful FMT results than the stool from other donors [133]. Typically, the FMT success is defined by a positive clinical outcome in the recipient [133]. However, how to predict the FMT success or find out a super-donor, particularly at a period prior to the implementation of FMT is still a challenging task. In addition, while the gut microbiome and the physio-pathological measurements of donor are considered as the predictors for FMT success [134], the FMT-microbes are finally located and shaped in the gut of recipients. With the better understanding of microbiota shaping factors, we will be able to elucidate the underlying mechanism of the microbiota formation in both donors and recipients. In donors, the microbial composition can be evaluated and linked to the host gene that is known to shape microbiota. In recipients, the survival and function of FMT-microbes can be predicted by evaluating the shaping factors existed in the gut of recipients. Moreover, the colonization efficacy of FMT-microbes can be predicted by matching some identified shaping factors between recipients and donors. The development of a panel of host genes associated with the host microbiota shaping would as a fast and efficient tool to predict FMT success in the future.

## Conclusion and perspective

In this review, we summarize the findings of the host factors that could shape the gut microbiota. While many evidences have showed that diet, nutrient, pharmacologic factors and many other stimuli are more dominant than host genetic factors in the modulation of gut microbial compositions [108, 135–137], it is inappropriate to ignore or exclude the impact of host genetic factors on the gut microbiota [25]. Conversely, the improvement of knowledge in particular how host factors shape the gut microbiota could provide the researchers more opportunities to manipulate the gut microbes, which has tremendous application potential in clinic and industry. Before that, more microbiome data in particular the microbiome genome-wide association studies (mGWAS) is required, and the artificial intelligence (AI) technology is considered as the new strategy for accelerating the analysis of the accumulated microbiome data. In addition, more knowledge from the mucus-based microbiota analysis is needed. Although the stool sample is relatively easy to collect, the microbe-host interactions mainly take place in the mucus layer [138]. Besides, the studies discussed in this review are mostly whole-body knockout of genes, and therefore further studies will be required to distinguish the epithelia-specific and the myeloid-derived effects. Finally, we should remind ourselves that the effect of hosts on the microbiota is not only contributed by one gene. The coordination between host genes should be taken into consideration to draw a complete map of host-microbe interaction.

## Data Availability

NA
